# Dietary fats promote functional and structural changes in the median eminence blood/spinal fluid interface—the protective role for BDNF

**DOI:** 10.1186/s12974-017-1046-8

**Published:** 2018-01-09

**Authors:** Albina F. Ramalho, Bruna Bombassaro, Nathalia R. Dragano, Carina Solon, Joseane Morari, Milena Fioravante, Roberta Barbizan, Licio A. Velloso, Eliana P. Araujo

**Affiliations:** 1 LAV, Laboratory of Cell Signaling, University of Campinas, Campinas, SP 13084-970 Brazil; 2 Faculty of Nursing, University of Campinas, Campinas, SP 13084-970 Brazil

**Keywords:** Obesity, Hypothalamus, Inflammation, Diet

## Abstract

**Background:**

The consumption of large amounts of dietary fats activates an inflammatory response in the hypothalamus, damaging key neurons involved in the regulation of caloric intake and energy expenditure. It is currently unknown why the mediobasal hypothalamus is the main target of diet-induced brain inflammation. We hypothesized that dietary fats can damage the median eminence blood/spinal fluid interface.

**Methods:**

Swiss mice were fed on a high-fat diet, and molecular and structural studies were performed employing real-time PCR, immunoblot, immunofluorescence, transmission electron microscopy, and metabolic measurements.

**Results:**

The consumption of a high fat diet was sufficient to increase the expression of inflammatory cytokines and brain-derived neurotrophic factor in the median eminence, preceding changes in other circumventricular regions. In addition, it led to an early loss of the structural organization of the median eminence β1-tanycytes. This was accompanied by an increase in the hypothalamic expression of brain-derived neurotrophic factor. The immunoneutralization of brain-derived neurotrophic factor worsened diet-induced functional damage of the median eminence blood/spinal fluid interface, increased diet-induced hypothalamic inflammation, and increased body mass gain.

**Conclusions:**

The median eminence/spinal fluid interface is affected at the functional and structural levels early after introduction of a high-fat diet. Brain-derived neurotrophic factor provides an early protection against damage, which is lost upon a persisting consumption of large amounts of dietary fats.

**Electronic supplementary material:**

The online version of this article (10.1186/s12974-017-1046-8) contains supplementary material, which is available to authorized users.

## Background

Body mass stability relies on a complex interaction between neurons that sense the energy status of the body and effector neurons that coordinate food intake and energy expenditure [1]. Most energy status-sensing neurons are located in the mediobasal hypothalamus and are set to respond to circulating hormones and nutrients that indicate the short- and long-term fluctuations in whole-body energy stores [1, 2]. In order to reach energy-sensing neurons, circulating factors must cross the median eminence (ME)/spinal fluid interface (SFI) [3, 4]. Recent studies have provided strong evidence to support an important role for ME tanycytes as gatekeepers that control the access of circulating factors to mediobasal neurons [5, 6].

In diet-induced obesity, long-chain saturated fats trigger a TLR4- and endoplasmic reticulum stress-dependent inflammatory response in the hypothalamus resulting in severe damage to the neurons that control food intake and energy expenditure [7–10]. In the short run, the damage inflicted to hypothalamic neurons results in leptin and insulin resistance and is reversible [11, 12]. However, upon prolonged exposure to dietary fats, neurons may undergo apoptosis and the reversibility of the obese status becomes unlikely [12–14]. Interestingly, studies have shown that the consumption of dietary fats predominantly affects the hypothalamus, sparing other brain regions [7, 11, 14]. In fact, hypothalamic inflammation in response to the consumption of dietary fats is a very premature phenomenon, which can be detected as early as 1 day after the introduction of a high-fat diet [14, 15]. Here, we first hypothesized that the ME-SFI is particularly sensitive to dietary fats, leading to an early exposure of the neurons of the mediobasal hypothalamus to potentially damaging circulating factors; to explore this hypothesis, we microdissected the main periventricular BBB regions, i.e., ME, vascular organ of lamina terminalis (OVLT), subfornical organ (SFO), and subcomissural organ (SCO), and evaluated the impact of the consumption of large amounts of dietary fats on the expression of structural and inflammatory markers; we also evaluated the expression of brain-derived neurotrophic factor (BDNF), which has an important trophic action in the blood-brain barrier (BBB) [16]. These experiments demonstrated that the ME presented the earliest changes in the expression of inflammatory markers and BDNF. Next, we performed a series of structural studies to determine the temporal evolution of changes in the integrity of the ME-BBB in response to the consumption of large amounts of dietary fats. As early as 1 week, the consumption of dietary fats led to changes in the integrity and architecture of the region of the ME-SFI. Finally, we evaluated the impact of the modulation of BDNF in the progression of diet-induced obesity and the integrity of the ME-SFI. We show that reducing BDNF increases diet-induced body mass gain and enhances the functional and structural disarrangements in the ME-SFI zone.

## Methods

### Experimental animals

**Fig. 1:**
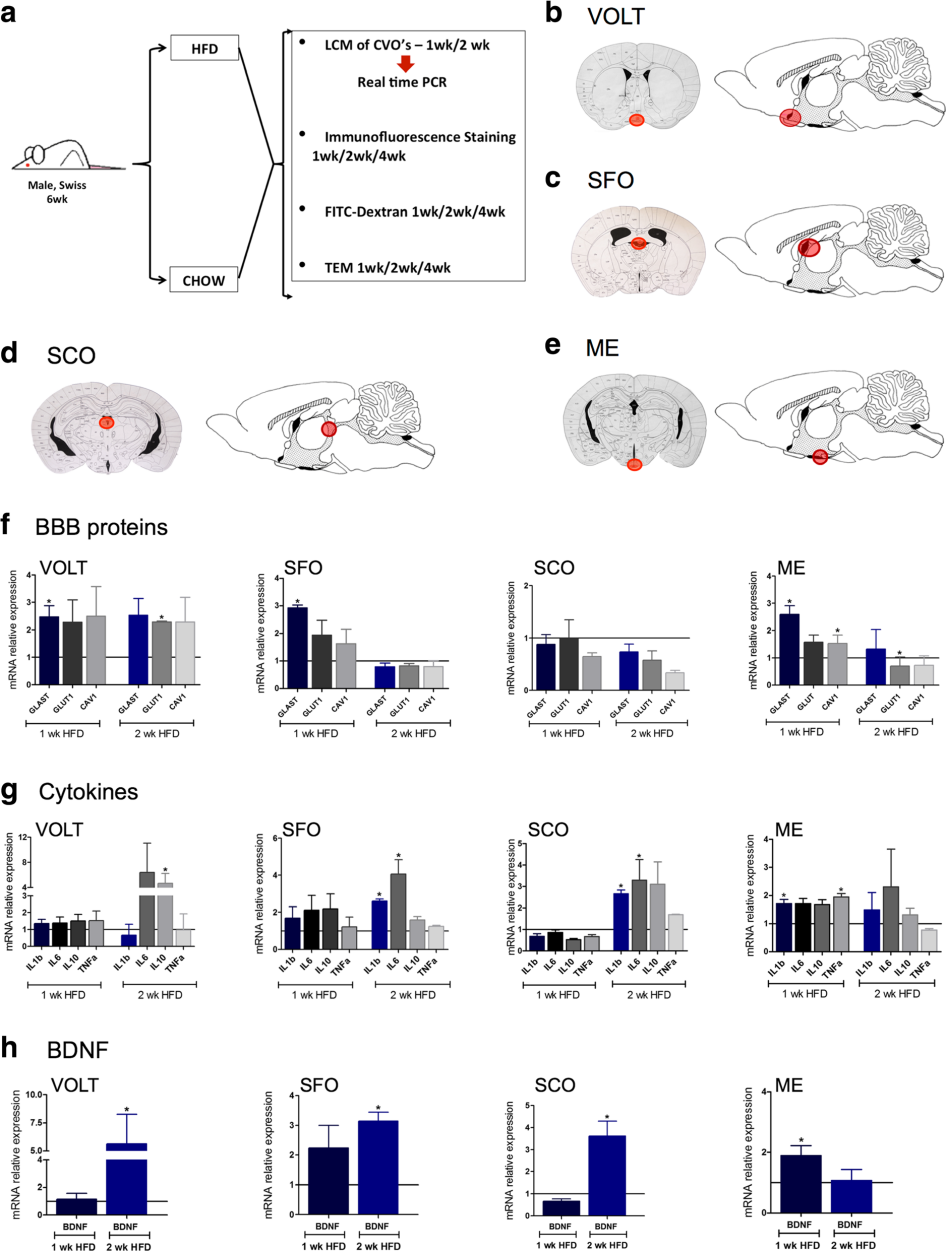
Evaluation of the impact of a high-fat diet on the expression of markers of blood-brain barrier, inflammation, and brain-derived neurotrophic factor in the circumventricular organs. In most of the experiments in this study, 6-week-old male Swiss mice were randomly divided to feed on chow or a high-fat diet (HFD) for 1, 2, or 4 weeks; at the end of the respective experimental periods, the mice were used in experiments: laser microdissection (LCM) of the circumventricular organs (CVO) for real-time PCR determination of specific transcripts, immunofluorescence staining of specific markers, FITC-dextran injection for evaluation of BBB integrity, and transmission electron microscopy (**a**). **b**–**e** schematic illustrations of the regions submitted to LCM for harvesting of specimens from CVOs. **f** Real-time determination of the relative expression of transcripts of markers of the BBB in the distinct CVOs. **g** Real-time determination of the relative expression of transcripts of cytokines in the distinct CVOs. **h** Real-time determination of the relative expression of transcript of BDNF in the distinct CVOs. In **f**–**h**, expression of target transcripts is presented as relative to paired controls fed chow (line in *y* = 1). In all experiments *n* = 4; *p < 0.05 vs. respective control. BBB blood-brain barrier, BDNF brain-derived neurotrophic factor, CVO circumventricular organs, HFD high-fat diet, LCM laser microdissection, ME median eminence, SCO subcomissural organ, SFO subfornical organ, TEM transmission electron microscopy, OVLT vascular organ of the lamina terminalis, wk week

Six-week-old male Swiss mice were kept in individual cages throughout the study, in standard photoperiod conditions (light/dark—12 h) at 22 ± 2 °C, and receiving diet and water ad libitum. For most experiments, mice were randomly divided into two groups fed either chow or a high-fat diet (HFD) for 1, 2, or 4 weeks (Fig. 1a). Chow was purchased from Nuvilab, São Paulo, Brazil, and the HFD was prepared at the lab by a certified nutritionist (the detailed composition of the diets is presented in Table 1). For some experiments, right after dietary intervention, mice were treated either with an immunoneutralizing rabbit anti-BDNF antibody (0.8 μg/100 μl, sc-546, Santa Cruz Biotechnology, Santa Cruz, CA, USA) or a pre-immune rabbit serum (#R9133, Sigma-Aldrich^®^, St. Louis, MO, USA), by intraperitoneal injection every 3 days for 2 or 4 weeks. In some experiments, mice were submitted to a protocol aimed at identifying obese-prone (OP) and obese-resistant (OR) animals; for that, 6-week-old Swiss mice were fed a HFD for 24 h and food intake was recorded. Mice were then grouped into quartiles for total food intake and only the top quartile (OP) and the bottom quartile (OR) mice were included in the experiments. In all experiments, we employed age-matched controls. Thereafter, OP and OR mice were submitted to a protocol for exogenous BDNF delivery (OP) or immunoneutralization of BDNF (OR), as described earlier in this paragraph. In all experiments, caloric intake and body mass were recorded. At the end of the experimental periods, mice were submitted to a 4-h fasting and then anesthetized with sodium thiopental, 50 mg/kg. The 4-h fasting protocol was employed because hypothalamic cells are sensitive to minimal nutrient variations; thus, in order to normalize experimental conditions, we tested the best fasting protocols to be employed in the experiments and the 4-h was elected. Once anesthesia was confirmed, mice were used in experiments for the collection of brain samples.

**Table 1:** Macronutrient composition of the diets

Components	Chow (Nuvilab CR1) (g)	High-fat diet (g)
Starch	427.5	115.5
Protein (casein 85%)	200	200
Dextrinized corn starch	132	132
Sucrose	100	100
Soybean oil	40	40
Lard	0	312
Fiber (cellulose)	50	50
Mineral mix (AIN-93)	35	35
Vitamin mix (AIN-93)	10	10
l-Cystine	3	3
Choline bitartrate	2.5	2.5
Total	1000	1000

### mRNA extraction and real-time PCR

The brains were rapidly excised, rinsed in a saline solution, frozen on dry ice, and stored at − 80 °C. Initially, thick (40 μm) brain sections were prepared in a cryostat (LEICA^®^ CM1520) to determine the correct locations of the target regions. Once identified, the target regions were submitted to laser capture microdissection using a LCM from PALM Robot Microbean (Carl-Zeiss, Gottingen, Germany). The anatomical landmarks of the distinct regions were always identified using the coordinates as described in the Paxinos Atlas of Stereotaxic Coordinates (Elsevier Science 2004). Specimens were immediately submitted to RNA extraction using the RNeasy Plus Micro Kit^®^ (Qiagen Sciences, Germantown, MD, USA) according to the manufacturer’s protocol. Because of the low levels of starting material, cDNA synthesis was conducted with total RNA obtained at extraction and using the Reverse Transcription High Capacity Reverse Transcription Kit (Applied Biosystems, Foster City, CA, USA). In another protocol, the brain was excised and the hypothalamus was removed and frozen at − 80 °C. Samples were homogenized in TRIzol^®^ (Invitrogen, São Paulo, Brazil) using a tissue homogenizer (Polytron-Aggregate, Kinematica, Littau/Luzern, Switzerland). Total RNA was isolated according to the manufacturer’s guidelines and quantified using NanoDrop^®^ (Synerge MX, BioTek^®^, Winooski, VT, USA). The integrity of RNA was evaluated by agarose gel electrophoresis. Complementary DNA was prepared using 2 μg of total RNA and reverse transcriptase. Subsequently (for both protocols), the cDNA was diluted depending on the concentration needed for efficient amplification of genes of interest. Real-time PCR reactions were performed using the TaqMan^®^ TM system (Applied Biosystems). Glyceraldehyde-3-phosphate dehydrogenase (GAPDH) was used as the endogenous control of the reaction, serving to normalize the expression of genes of interest in different samples. The genes analyzed were GLAST (Mm00600697_m1), GLUT1 (Mm00441480_m1), CAVEOLIN1 (Mm00483057_m1), TNFα (Mm00443258_m1), IL1β (Mm00434228_m1), IL6 (Mm00446190_m1), IL10 (Mm01288386_m1), BDNF (Mm01334043_m1), and CLAUDIN5 (MmPT5833394738.g) for the first protocol, plus GFAP (Mm01253033_m1), IGFBP2 (Mm00805581), NGFR (Mm.PT.58.10419996), and TrkB (Mm.PT.5842284287) added for the second protocol. For the relative quantification of genes under study, real-time PCR reactions were performed in triplicate from 3 μl TaqMan Universal PCR Master Mix 2X, 0.25 μl of primers and probe solution, 2.75 μl water, and 4.0 μl cDNA. The cycling conditions were 50 °C for 2 min and 95 °C for 10 min and 45 cycles of 95 °C for 15 s and 60 °C for 1 min. The values of relative gene expression were obtained by analyzing the results in the programme 7500 System SDS software (Applied Biosystems).

### Blood-brain barrier leakage analysis

Male mice were anesthetized, and fluorescein isothiocyanate dextran (FITC-dextran—2000 kDa, 100 μl, 50 mg/ml, in saline, Sigma-Aldrich, St. Louis, MO) was injected intravenously in the inferior vena cava vein. After 1 min, the brains were removed, rinsed with saline, and fixed in 4% paraformaldehyde for 24 h; thereafter, the specimens were cryoprotected in 30% sucrose for 48 h and then submitted to coronal sectioning (20 μm thickness) using the cryostat (Leica Microsystems, CM1860, Buffalo Grove, USA). Fluorescent images of the sections were obtained using a confocal fluorescence microscope (Leica) always employing the same parameters for acquiring images (laser 488, wavelength = 405, %laser = 20%, gain = 1015, offset = − 0.3799). Regional FITC-dextran fluorescent intensity was measured by ImageJ (National Institute of Health, Bethesda, MD). Optical density was measured by applying the following formula: (right fluorescent intensity − left fluorescent intensity)/left fluorescent intensity × 100.

### Immunohistochemistry

Mice were perfused with 4% paraformaldehyde, and the whole brain was removed and submitted to 24-h fixation in 4% paraformaldehyde. Thereafter, the brain was rinsed in 1× PBS and cryoprotected in 30% sucrose for 48 h and then submitted to coronal sectioning (20 μm thickness) using the cryostat (LEICA Microsystems^®^, CM1860, Buffalo Grove, IL, USA). Sections were rinsed in 0.1 M PBS (pH 7.4) and blocked for 1 h at room temperature in a blocking solution (5% normal goat serum, 0.2% Tween 80^®^ in PBS). The slides were incubated overnight at 4 °C with either anti-IGFBP2 (sc-365368, 1:200, Santa Cruz Biotechnology, Dallas, TX, USA), anti-FGF10 (ABN44, 1:200, Merck Millipore, Temecula, CA, USA), anti-vimentin (sc-373717, 1:200, Santa Cruz Biotechnology, Dallas, TX, USA), anti-GFAP (ab7260, 1:1000, ABCAM, Cambridge, UK), anti-BDNF (sc-546, 1:200, Santa Cruz Biotechnology, Dallas, TX, USA), or anti-TrkB (sc-8316, 1:200, Santa Cruz Biotechnology, Dallas, TX, USA) in a blocking solution (1% bovine serum albumin), then for 1 h with goat Anti-Mouse FITC^®^ (sc-2010, 1:200, Santa Cruz Biotechnology, Dallas, TX, USA) and goat Anti-rabbit Cy3^®^ (ab6941, ABCAM, 1:500, ABCAM, Cambridge, UK) in a blocking solution, and the nuclei were stained with (TO-PRO^®^-3 Iodide (642/661), T3605, 1:1000, Life Technologies, Carlsbad, CA, EUA) in PBS. The specificity of the antibodies have been tested either by others [17–20] or by us, by performing immunodepletion experiments. Negative controls were performed by omitting the first antibody. In all experiments, 3–4 mice were analyzed in each group; at least four distinct sections from each mouse hypothalamus were evaluated. The sections were examined by fluorescence microscopy using a confocal microscope LEICA TCS SP5 II (Leica). Regional fluorescent intensity was measured by ImageJ (National Institute of Health, Bethesda, MD). Optical density was measured by applying the following formula: (right fluorescent intensity − left fluorescent intensity)/left fluorescent intensity × 100. All fluorescence images were quantified, and the means ± SD are presented in Additional files 1, 2, 3, 4, 5, and 6: Tables S1–S6.

### Electron microscopy

Mice were perfused with Karnowsky reagent, and the whole brain was removed and prepared for transmission electron microscopy analysis as previously described [12].

### Body composition by PET/CT

Body composition was obtained by positron emission tomography/CT scan (Albira/Bruker, Billerica, MA, USA) as previously described [21].

### Statistics analysis

Results are presented as the means ± standard deviation (SD). For statistical analysis, first, we applied the Levene test to check the homogeneity of variances. For the comparison of means between two groups, we used Student’s *t* test for independent samples. The significance level to reject the null hypothesis was set at *p* < 0.05. One-way or two-way ANOVA was used to evaluate the experiments with treatment with anti-BDNF. The level of significance was set at *p* < 0.05. The data were analyzed using the GraphPad Prism^®^.

## Results

### The median eminence is the circumventricular organ affected the earliest in response to the consumption of a HFD

Mice fed chow or HFD for 1 or 2 weeks were used in experiments aimed at microdissecting the circumventricular organs, OVLT (Fig. 1b), SFO (Fig. 1c), SCO (Fig. 1d), and ME (Fig. 1e). The microdissected specimens were used in real-time PCR experiments for determining the expression of transcripts encoding structural proteins of the BBB, GLAST, GLUT1, and caveolin-1 (Fig. 1f); the transcripts encoding cytokines, IL1β, IL6, IL10, and TNFα (Fig. 1g); and the transcript encoding BDNF (Fig. 1h). The earliest changes induced by the consumption of a HFD occurred after 1 week were as follows: GLAST was increased in OVLT, SFO, and ME and caveolin-1 was increased only in ME (Fig. 1f); IL1β and TNFα (Fig. 1g) as well as BDNF (Fig. 1h) were increased only in ME. Interestingly, after 2 weeks on a HFD, the expression of BDNF was normalized in ME, whereas in all the remaining regions evaluated, its expression increased (Fig. 1h). Because major changes occurred in ME, we measured transcript expression of claudin-5, a tight junction protein. As shown in Additional file 7: Figure S1, in ME, claudin-5 expression was reduced after 1 week on a HFD, returning to normal levels after 2 weeks and reducing again after 4 weeks.

### The consumption of a HFD leads to an early loss of integrity in the ME-BBB

**Fig. 2:**
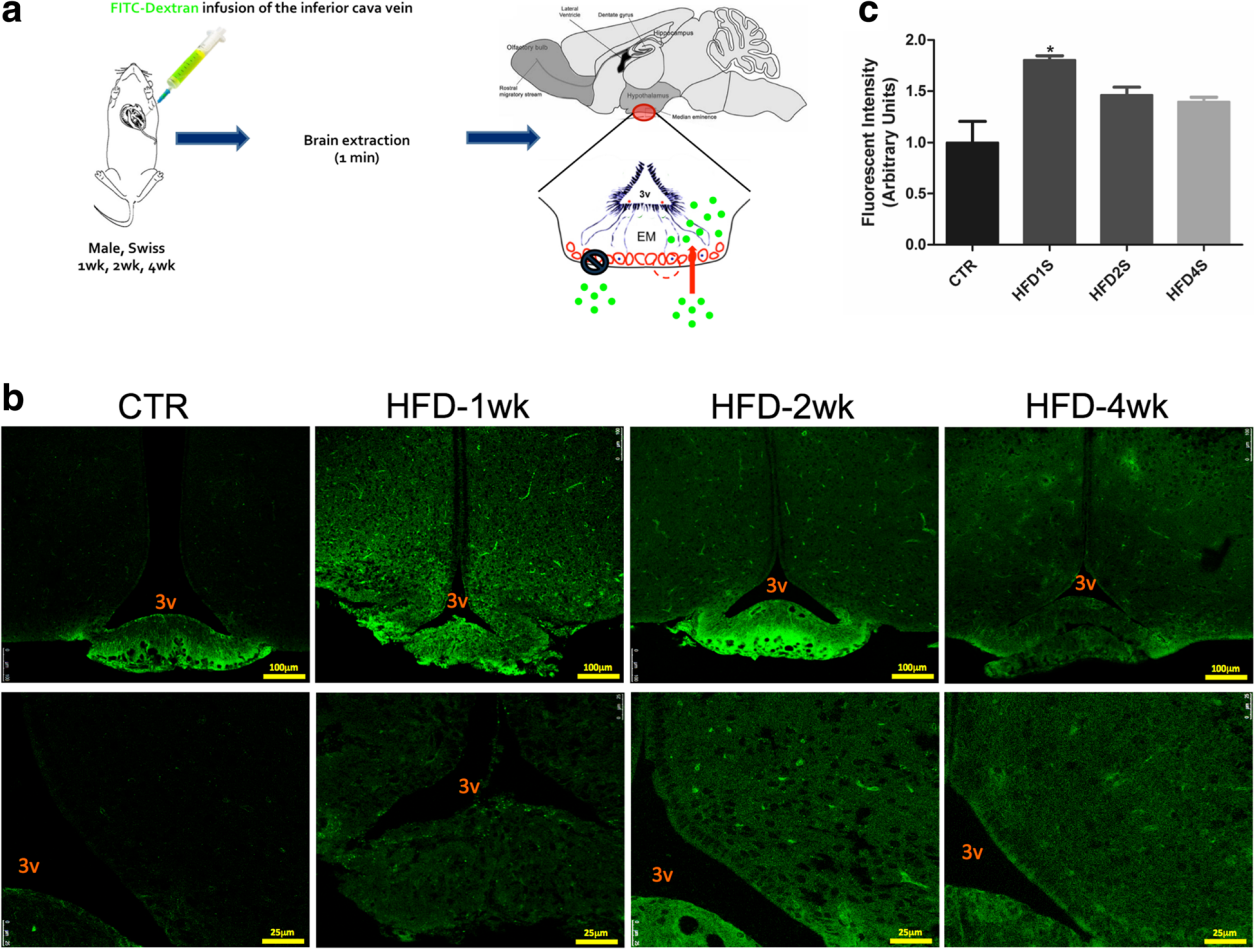
Evaluation of median eminence blood-brain barrier integrity. **a** Schematic representation of the protocol employed for evaluation of BBB integrity; in the enlarged scheme of the median eminence (ME) (right hand, bottom), FITC-dextran is shown as small green circles; in the case of barrier integrity, FITC-dextran is retained out of ME, whereas the loss of integrity allows transposition of FITC-dextran into the ME and neighboring areas. **b** Confocal microscopy analysis of FITC-dextran endogenous fluorescence in the region of the ME; in all acquisitions, the same settings of the microscope were employed (laser 488, wavelength = 405, %laser = 20%, gain = 1015, offset = − 0.3799). **c** Fluorescence in the ME was determined using an ImageJ software and presented as relative to control. *N* = 4; *p < 0.05 vs. control. 3v third ventricle, CTR control fed chow, HFD high-fat diet, ME median eminence, wk week

**Fig. 3:**
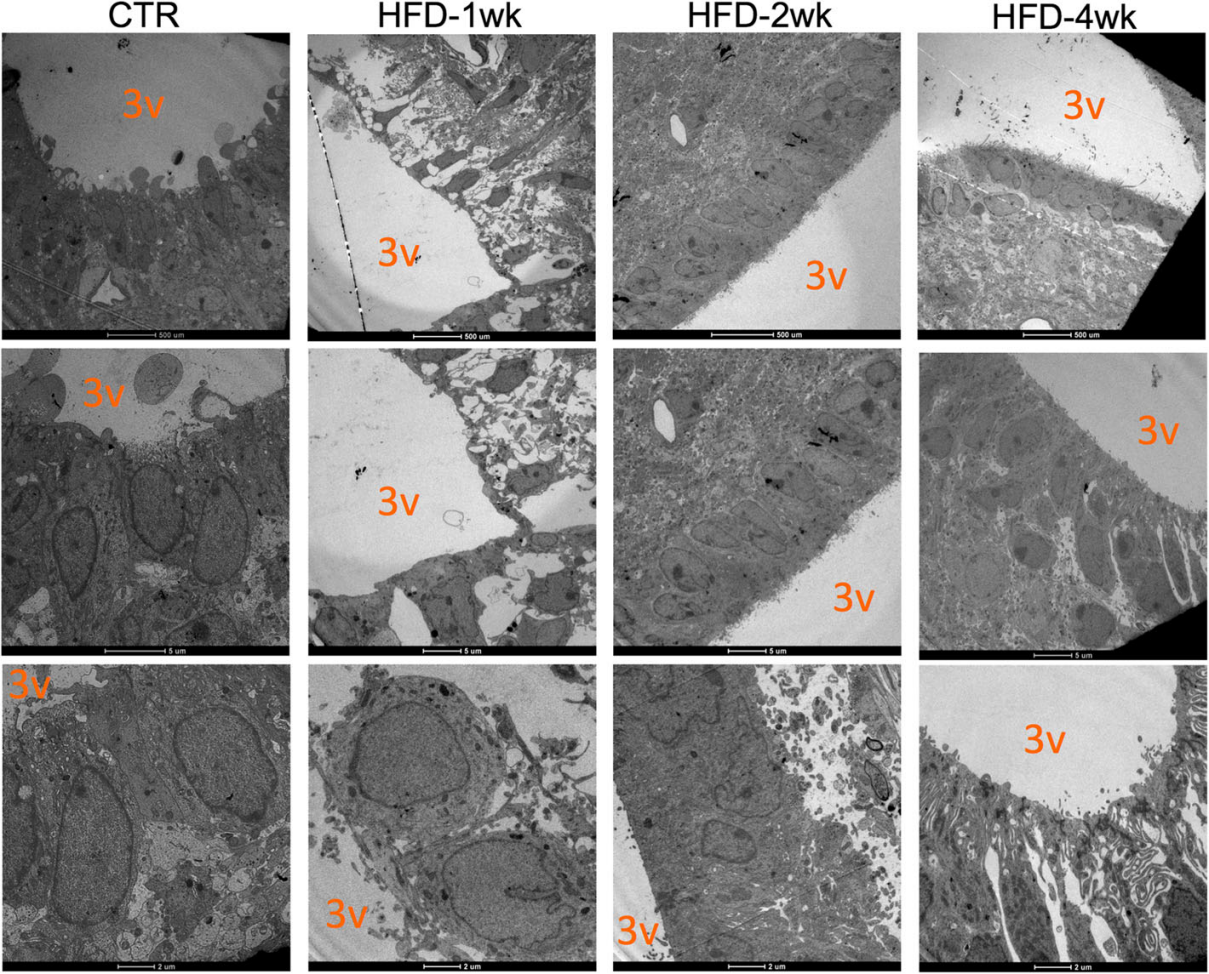
Ultrastructural analysis of the cells in the interface between the median eminence, arcuate nucleus, and third ventricle. Transmission electron microscopy images depicting the region of the interface between the median eminence, arcuate nucleus, and third ventricle. The images are representative of three independent experiments. 3v third ventricle, CTR control fed chow, HFD high-fat diet, wk week

In order to determine if the early changes in the expression of structural, inflammatory, and neurotrophic proteins in the ME were accompanied by changes in BBB permeability, mice were treated with dextran-fluorescein (Fig. 2a) and the integrity of the barrier was evaluated by confocal microscopy. As depicted in Fig. 2b and quantified in Fig. 2c, as early as 1 week after the introduction of a HFD, there was a significant increase in fluorescence in the ME and surrounding areas of the mediobasal hypothalamus. No changes in permeability were detected in OVLT, SFO, and SCO (Additional file 8: Figure S2). Next, we employed transmission electron microscopy to evaluate the details of the cellular organization in the region of the ME. As depicted in Fig. 3, as early as 1 week after the introduction of a HFD, there was a major change in the shapes of the cell lining in the limits of the third ventricle, neighboring the ME. In mice fed chow, the cells were elongated, with a homogenous aspect of the cytoplasm, perfectly aligned and juxtaposed with very little extracellular spacing. After 1 week on a HFD, the shapes of the cells were changed, becoming heterogeneous with some cells assuming an even more elongated shape (as in the top panel), whereas others becoming cuboidal (as in the bottom panel); in addition, the aspect of the cytoplasm was heterogeneous and there was a large extracellular spacing. Interestingly, in mice fed a HFD for 2 weeks, the aspect of the cells was very similar to the one described for mice fed chow, whereas in mice fed a HFD for 4 weeks, cell shape and tissue organization were again altered, similar to the changes described for mice fed a HFD for 1 week.

**Fig. 4:**
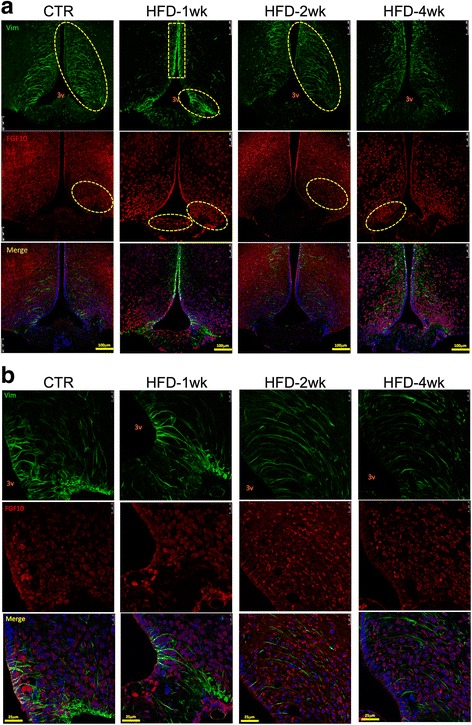
Immunofluorescence staining of markers of the median eminence blood-brain barrier I. **a** Low magnification and **b** high magnification of immunofluorescence staining using primary antibodies against vimentin (green) and FGF10 (red). Specimens were obtained from bregma-anteroposterior − 2.06 to − 2.18. The images are representative of three independent experiments. 3v third ventricle, CTR control fed chow, FGF10 fibroblast growth-factor 10, HFD high-fat diet, wk week, Vim vimentin. The main histological findings described in the text are highlighted by the yellow dashed boxes/ellipses

**Fig. 5:**
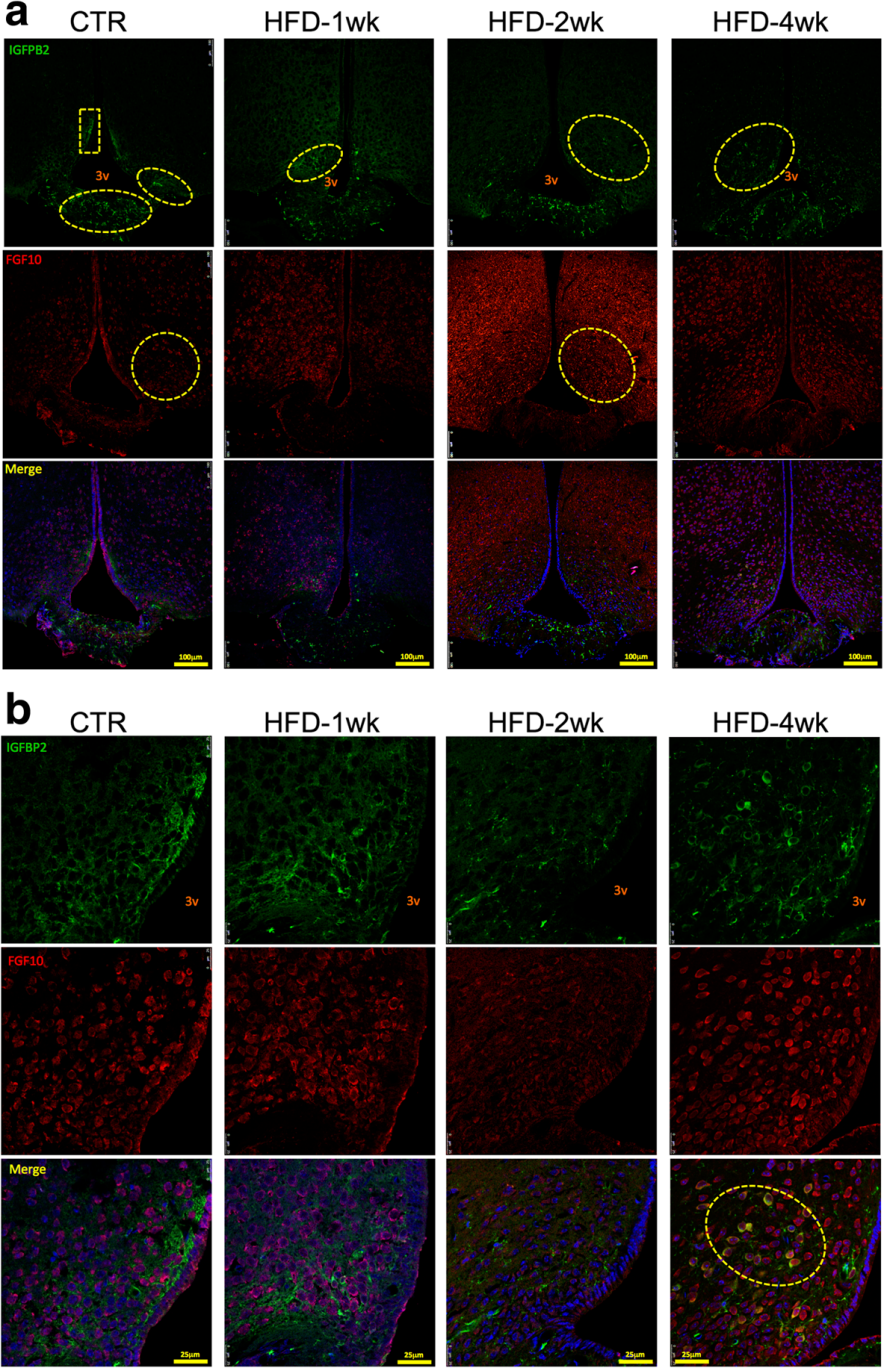
Immunofluorescence staining of markers of the median eminence blood-brain barrier II. **a** Low magnification and **b** high magnification of immunofluorescence staining using primary antibodies against IGFBP2 (green) and FGF10 (red). Specimens were obtained from bregma-anteroposterior − 2.06 to − 2.18. The images are representative of three independent experiments. 3v third ventricle, CTR control fed chow, FGF10 fibroblast growth factor 10, HFD high-fat diet, IGFBP2 insulin-like growth factor-binding protein 2, wk week. The main histological findings described in the text are highlighted by the yellow dashed boxes/ellipses

**Fig. 6:**
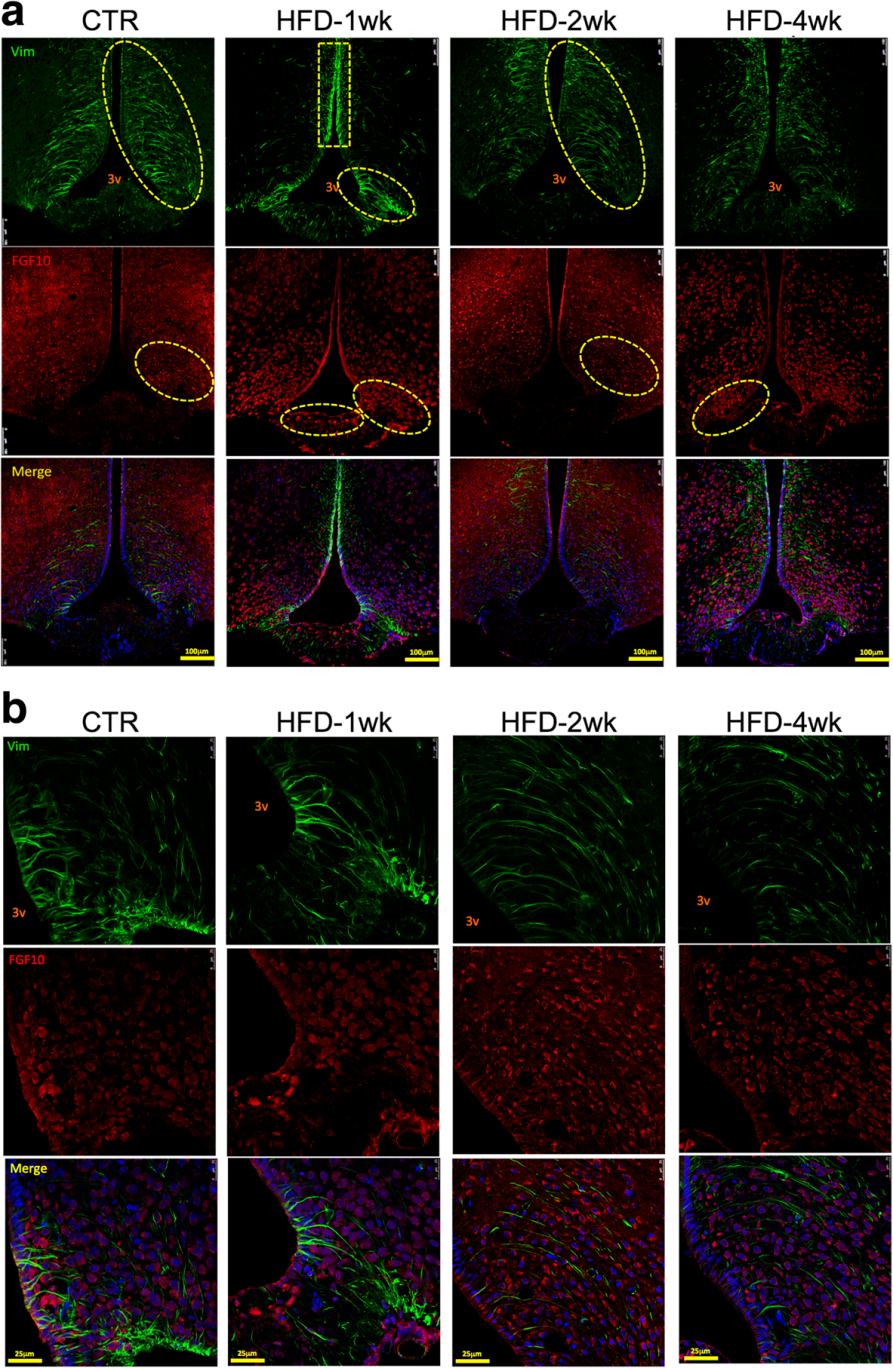
Immunofluorescence staining of markers of the median eminence blood-brain barrier III. **a** Low magnification and **b** high magnification of immunofluorescence staining using primary antibodies against IGFBP2 (green) and GFAP (red). Specimens were obtained from bregma-anteroposterior − 2.06 to − 2.18. The images are representative of three independent experiments. 3v third ventricle, CTR control fed chow, GFAP glial fibrillary acidic protein, IGFBP2 insulin-like growth factor-binding protein 2, HFD high-fat diet, wk week. The main histological findings described in the text are highlighted by the yellow dashed ellipses

In order to further explore the structural changes induced by the consumption of a HFD in the ME, we used immunofluorescence staining to evaluate markers of tanycytes and other proteins that play important roles in the integrity of the SFI and BBB. As depicted in Fig. 4, the expression and distribution of vimentin and FGF10 underwent major changes in the hypothalamus of mice fed a HFD for 1 week, returning to a morphology similar to control at 2 weeks, and finally becoming altered again after 4 weeks. In control and 2 weeks on a HFD, vimentin was present in a radial aspect emanating from the third ventricle, whereas in mice fed a HFD for 1 week, it was predominantly present in the upper part of the walls of the third ventricle and in the junction between the ME and the arcuate nucleus. In control and mice fed a HFD for 2 weeks, FGF10 was virtually absent from cells in the ME, whereas most of its expression occurred in cells spread though the mediobasal hypothalamus. In mice fed a HFD for 1 or 4 weeks, FGF10 was predominantly expressed in the transition between the ME and the arcuate nucleus, and, particularly, in mice fed a HFD for 1 week, there was FGF10 in a number of cells in the ME. There were no cells co-expressing vimentin and FGF10 in the groups evaluated. IGFBP2 is regarded as an important marker of the β1-tanycytes [22, 23]. In Fig. 5, we show that in control mice, its expression is present in cells in the middle portion of the walls of the third ventricle, in the ME, and in the transition between the ME and the arcuate nucleus. Following the beginning of feeding on a HFD, the expression of IGFBP2 changed rapidly, disappearing from the walls of the third ventricle, decreasing in the region of transition between the ME and the arcuate nucleus, and increasing in cells scattered through the mediobasal hypothalamus. These changes were present and increased continuously from week 1 to week 4 after the introduction of a HFD. In control and in mice fed a HFD for 1 and 2 weeks, there was no co-expression of IGFBP2 and FGF10; however, in mice fed a HFD for 4 weeks, there was intense co-expression of IGFBP2 and FGF10 in cells in the mediobasal hypothalamus, as magnified in the panels in the bottom of Fig. 5b. Because of the change in distribution of cells expressing IGFBP2, we evaluated its co-expression with GFAP, a marker of astrocytes. As depicted in Fig. 6, there was no co-expression of IGFBP2 and GFAP in control and mice fed a HFD for 1 or 2 weeks; however, in week 4 after the introduction of a HFD, astrocytes increased in numbers in the mediobasal hypothalamus, and there were some cells co-expressing IGFBP2 and GFAP, as magnified in the panels in the bottom of Fig. 6b. The specific fluorescence of images obtained in the experiments presented in Figs. 4, 5, and 6 were quantified and are presented as Additional files 1, 2, 3, 4, 5, and 6: Tables S1–S6. Despite the fact that some of the quantifications revealed no significant differences, it is important to consider that the distribution rather than the amount of proteins may be more important in the context of the integrity of the barrier.

### The consumption of a HFD affects the expression of proteins of the BDNF system in the region of the ME-SFI

**Fig. 7:**
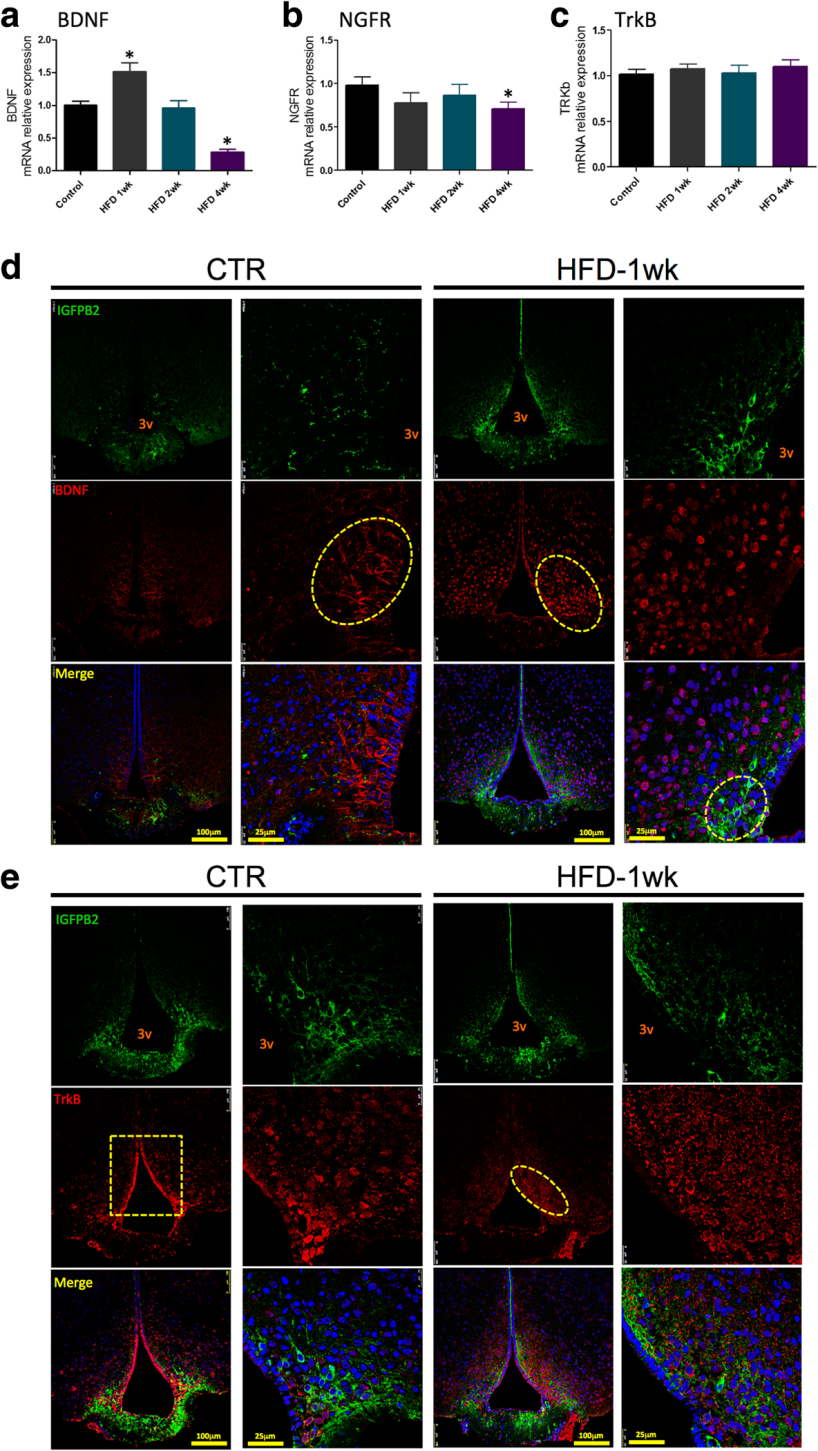
Hypothalamic expression of proteins of the brain-derived neurotrophic factor system in mice fed a high-fat diet. **a**–**c** Real-time PCR determination of the expression of transcripts encoding for BDNF (**a**), NGFR (**b**), and TrkB (**c**) in the median eminence of mice. **d**, **e** Immunofluorescence staining using primary antibodies against IGFBP2 (green) and TrkB (red). Specimens were obtained from bregma-anteroposterior − 2.06 to − 2.18. The images are representative of three independent experiments. In **a**–**c**, *n* = 4; *p < 0.05 vs. respective control. 3v third ventricle, BDNF brain-derived neurotrophic factor, CTR control fed chow, IGFBP2 insulin-like growth factor-binding protein 2, HFD high-fat diet, NGFR nerve growth factor receptor, wk week. The main histological findings described in the text are highlighted by the yellow dashed boxes/ellipses

The hypothalamic expression of BDNF increases after 1 week on a HFD, returning to normality at 2 weeks and undergoing a significant reduction after 4 weeks on a HFD (Fig. 7a). The expression of NGFR, the BDNF receptor involved in the induction of apoptosis, undergoes a discrete but significant reduction in the hypothalamus of mice fed a HFD for 4 weeks (Fig. 7b). The consumption of a HFD promoted no changes in the expression of TrkB, the BDNF receptor involved in neuroprotection (Fig. 7c). In immunofluorescence staining experiments, we observed that in control mice, BDNF is expressed in cytoplasmic projections of cells emanating from the lateral walls of the third ventricle (Fig. 7d). One week after the introduction of a HFD, there is an apparent increase in the number of cells expressing BDNF, which appears to localize predominantly in the peri-nuclear region of cells distributed through the mediobasal hypothalamus. Although most cells expressing BDNF do not express IGFBP2, there are some cells near the angle of the third ventricle and in the region of interface between the ME and the arcuate nucleus that co-express these two proteins (Fig. 7d, high magnification/merge). The distribution of TrkB is also affected by the consumption of a HFD (Fig. 7e). Thus, in control mice, most of the cells expressing TrkB are present in the walls of the third ventricle; upon feeding on a HFD for 1 week, the expression of TrkB along the wall of the third ventricle is much reduced, and most cells expressing this receptor are distributed within the mediobasal hypothalamus (Fig. 7e). There was no evidence of co-expression of TrkB and IGFBP2 (Fig. 7e).

### The immunoneutralization of BDNF increases diet-induced body mass gain, intensifies hypothalamic inflammation, and intensifies the structural disorganization of the ME-SFI region

**Fig. 8:**
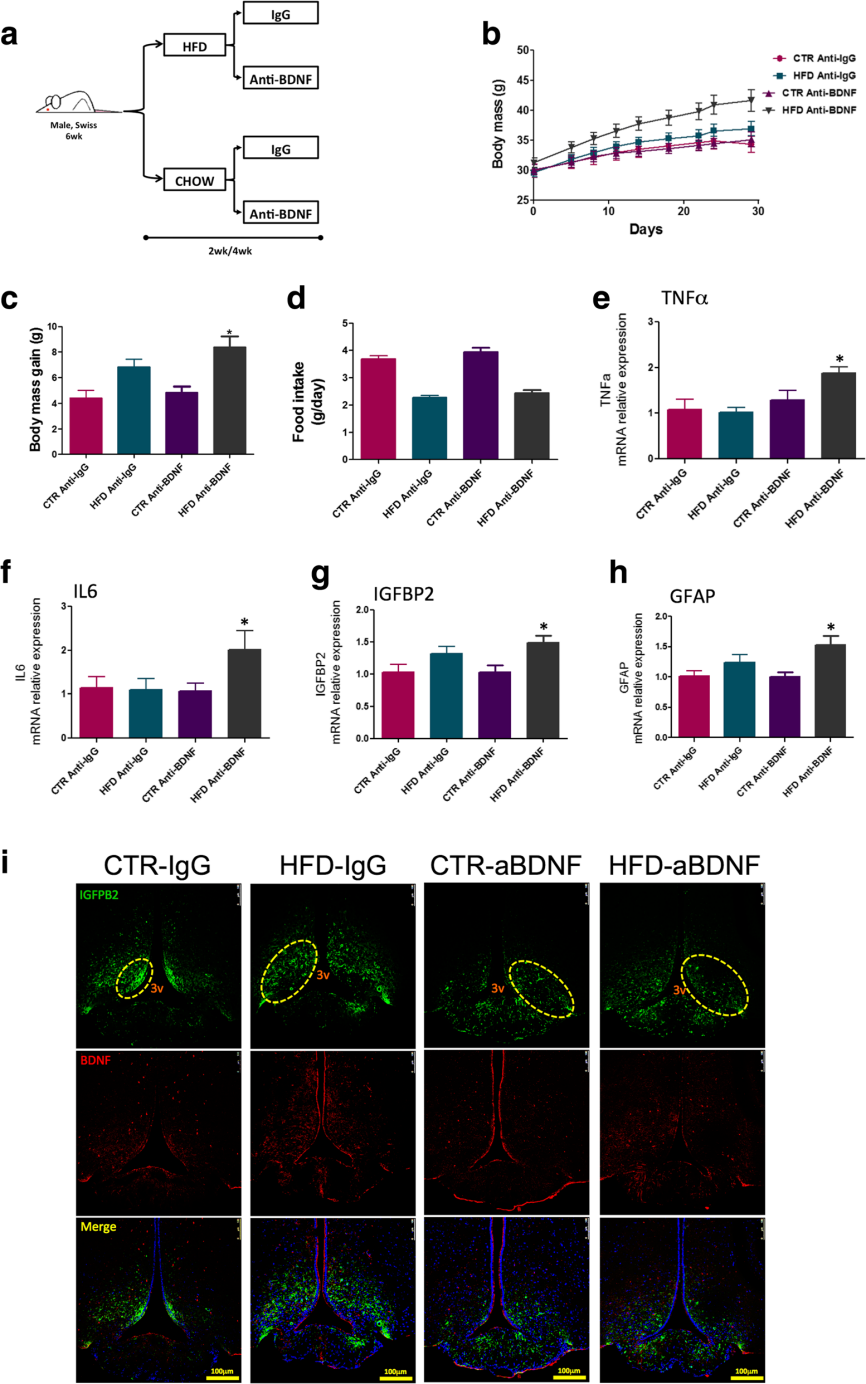
Immunoneutralization of BDNF. Schematic representation of the protocol employed to immunoneutralize BDNF (**a**). **b** Body mass during the experimental period. **c** Body mass variation during the experimental period. **d** Mean daily food intake during the experimental period. **e**–**h** Real-time PCR determination of the expression of transcripts encoding for TNFα (**e**), IL6 (**f**), IGFBP2 (**g**), and GFAP (**h**) in the median eminence of mice. **i** Immunofluorescence staining using primary antibodies against IGFBP2 (green) and BDNF (red). Specimens were obtained from bregma-anteroposterior − 2.06 to − 2.18. The images are representative of three independent experiments. In **e**–**h**, *n* = 4; *p < 0.05 vs. respective control. 3v third ventricle, aBDNF treated with immunoneutralizing antibody against BDNF, BDNF brain-derived neurotrophic factor, CTR control fed chow, GFAP glial fibrillary acidic protein, IGFBP2 insulin-like growth factor-binding protein 2, HFD high-fat diet, wk week. The main histological findings described in the text are highlighted by the yellow dashed boxes/ellipses

In order to evaluate the importance of BDNF in hypothalamic inflammation induced by a HFD, mice were treated for 2 or 4 weeks with an immunoneutralizing antibody against BDNF and metabolic and inflammatory parameters were measured. The capacity of immunoneutralizing antibodies injected systemically to act in the brain has been tested with success in other studies [24]. Figure 8a depicts the protocol employed in the experiments. As shown in Fig. 8b, c, the immunoneutralization of BDNF resulted in an increased body mass gain, without affecting caloric intake (Fig. 8d). This was accompanied by increased expression of TNFα (Fig. 8e) and IL6 (Fig. 8f) in the hypothalamus. In addition, there were increases in the expressions of IGFBP2 (Fig. 8g) and GFAP (Fig. 8h) (Additional file 9: Figure S3A). In immunofluorescence staining (Fig. 8i), the distribution of cells expressing IGFBP2 was modified by the inhibition of BDNF. In mice fed on chow, instead of its preferential localization in the lateral walls of the third ventricle (control mice), the inhibition of BDNF resulted in the spreading of IGFBP2-expressing cells in ME and throughout the mediobasal hypothalamus. In mice fed a HFD, the disorganized distribution of IGFBP2-expressing cells is further intensified following BDNF immunoneutralization. This was accompanied by increased barrier permeability in the region of the ME (Additional file 10: Figure S4). There was some modification in the pattern of expression and distribution of IBA1 in the hypothalamus of mice treated with the BDNF immunoneutralizing antibody, which was present in a larger number of cells present in the medial part of the arcuate nucleus nearby the limits of the median eminence (Additional file 9: Figure S3).

### The immunoneutralization of BDNF increases body mass gain and worsens diet-induced hypothalamic inflammation in obesity-resistant mice

**Fig. 9:**
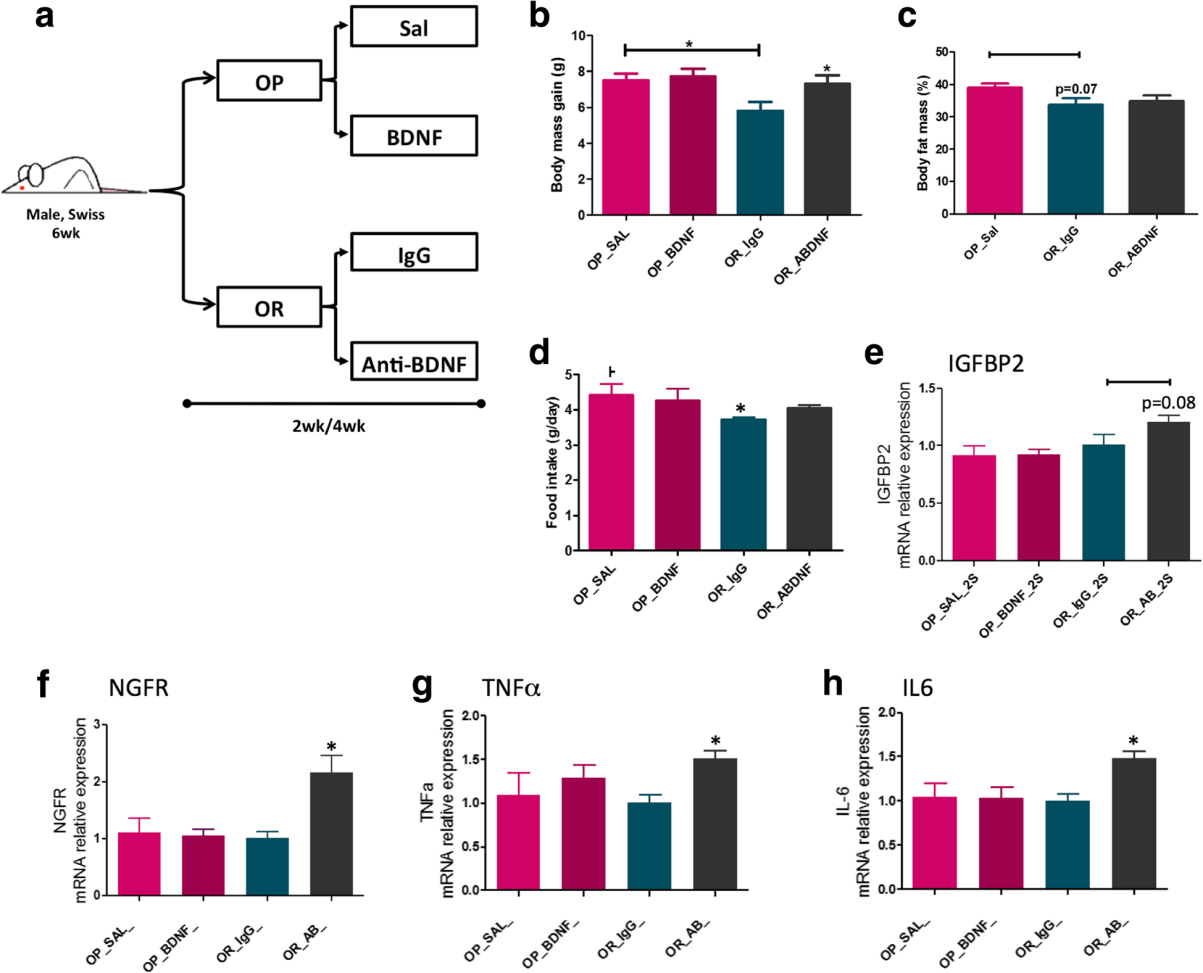
Modulating BDNF in obese-prone and obese-resistant mice. Schematic representation of the protocol employed to modulate BDNF in obese-prone (OP) and obese-resistant (OR) mice (**a**). Body mass variation (**b**), percent fat mass as determined by PET-CT scan (**c**), and mean daily food intake (**d**) in the experimental models. In **e**–**h**, real-time PCR determination of the expression of transcripts encoding for IGFBP2 (**e**), NGFR (**f**), TNFα (**g**), and IL6 (**h**) in the median eminence of mice. In **e**–**h**, *n* = 4; *p < 0.05 vs. respective control. aBDNF treated with immunoneutralizing antibody against BDNF, BDNF brain-derived neurotrophic factor, IGFBP2 insulin-like growth factor-binding protein 2, IL6 interleukin-6, NGFR nerve growth factor receptor, Sal saline, TNFa tumor necrosis factor-alpha, wk week

**Fig. 10:**
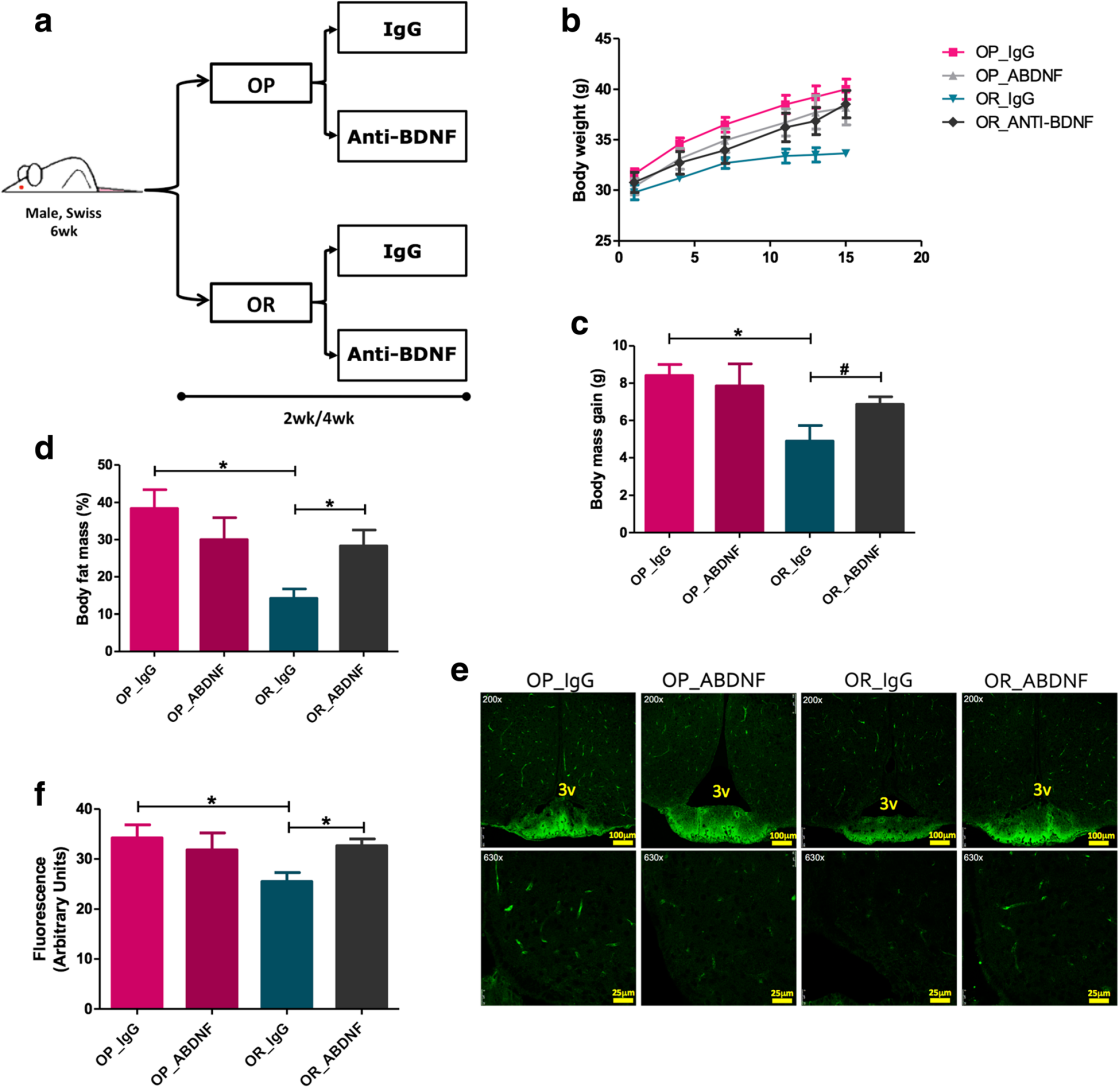
Immunoneutralization of BDNF in obese-prone and obese-resistant mice. Schematic representation of the protocol employed to immunoneutralize BDNF in obese-prone (OP) and obese-resistant (OR) mice (**a**). Body mass gain (**b**), body mass variation (**c**), and percent fat mass as determined by PET-CT scan (**d**) during the experimental period. **e** Confocal microscopy analysis of FITC-dextran endogenous fluorescence in the region of the ME; in all acquisitions, the settings of the microscope were employed (laser 488, wavelength = 405, %laser = 20%, gain = 1015, offset = − 0.3799). **f** Fluorescence in the ME was determined using an ImageJ software and presented as relative to control. In all experiments, *n* = 4; *p < 0.05. 3v third ventricle, aBDNF treated with immunoneutralizing antibody against BDNF, IgG treated with pre-immune IgG, wk week

Swiss mice are an outbred strain that, like humans, present a normal distribution of body mass gain whenever fed a high-fat diet [25]. Here, we took advantage of this fact to evaluate the impact of changing hypothalamic BDNF levels in OP and OR mice. First, we hypothesized that exogenous BDNF administration in the hypothalamus could protect OP mice from diet-induced obesity, whereas immunoneutralization of BDNF could increase diet-induced body mass gain of OR mice. To test this hypothesis, mice were submitted to a protocol as depicted in Fig. 9a and metabolic, structural, and inflammatory parameters were evaluated. As a whole, the treatment of OP mice with exogenous BDNF resulted in no change in body mass (Fig. 9b), in caloric intake (Fig. 9c), or in the expression of IGFBP2 (Fig. 9d), NGFR (Fig. 9e), TNFα (Fig. 9f), and IL6 (Fig. 9g). Conversely, the immunoneutralization of BDNF in OR mice promoted body mass gain (Fig. 9b) leading to increased adiposity (Fig. 9c), which was accompanied by increased caloric intake (Fig. 9d), therefore reverting the natural protection against diet-induced obesity in this subset of mice. This was accompanied by a trend to increase hypothalamic IGFBP2 (Fig. 9e) and to significantly increase NGFR (Fig. 9f), TNFα (Fig. 9g), and IL6 (Fig. 9h). Next, when both OP and OR mice were treated with an immunoneutralizing antibody against BDNF (Fig. 10a), body mass increase was observed in OR mice only (Fig. 10b, c). This was accompanied by increased whole-body adiposity (Fig. 10d) and by an increased leakage of the ME-SFI (Fig. 10e, f).

## Discussion

The main objective of this study was to evaluate the impact of the consumption of a HFD on the structural organization and integrity of the ME-SFI. Studies have shown that the consumption of large portions of dietary fat can trigger an inflammatory response in the hypothalamus leading to an impaired capacity of hypothalamic neurons to control caloric intake and energy expenditure, resulting in a progressive increase of body adiposity (this theme was revised previously [26]). Interestingly, there is certain specificity in diet-induced inflammation in the central nervous system since regions other than the hypothalamus either are not affected or, if so, are affected in much lower magnitude, and later than the hypothalamus [11, 14, 27].

Fatty acids present in the bloodstream are not freely diffusible into the brain [28]. Under certain conditions, they rely on specific transport systems present in the structures of the BBB [29, 30]. The appropriate function and distribution of these transport systems is of major importance in brain development and physiology throughout life because most fatty acids that constitute the central nervous system phospholipids cannot be synthetized de novo in the brain and thus must be imported from the periphery [28, 30]. Regarding the hypothalamus, its function as a nutrient sensor [31, 32] may explain why the ME-BBB presents some degree of permissiveness to certain nutrients, including fatty acids [31, 32]. In fact, a number of studies have explored the roles of fatty acids to control the function of hypothalamic neurons, implying that under physiological conditions, they are readily available [31]. Nevertheless, despite the fact that under physiological conditions and under the consumption of diets containing nutritionally adequate amounts of fat (such as in regular chow for rodents), there is some leakiness of the ME-BBB to fatty acids; the increased consumption of dietary fats may be capable of further increasing the BBB permeability to the point that the composition of fatty acids in the hypothalamus can be modified by dietary approaches [33, 34]. With these concepts in mind, in the first part of the study, we asked if the consumption of large portions of dietary fats would act differently in the distinct CVOs to promote changes in the expression of proteins involved in the structure of the BBB, cytokines, and BDNF. For that, we microdissected OVLT, SFO, SCO, and ME and evaluated the expression of target transcripts by real-time PCR. Except for GLAST that was increased in OVLT, SFO, and ME after 1 week on HFD, all the other transcripts undergoing rapid (1 week) induction by the dietary approach were increased in ME only. There were increases of transcripts encoding proteins of the BBB (GLAST and caveolin-1), cytokines (IL1β and TNFα), and BDNF. Thus, we concluded that the ME is particularly and rapidly responsive to the presence of high amounts of fatty acids in the diet, a fact that may contribute to a diet-induced dysfunction of the BBB at this anatomical location. It is noteworthy that changes in the expression of both inflammatory markers and BBB-related transcripts occurred in a biphasic manner. This is in consensus with the studies that evaluated details of diet-induced hypothalamic inflammation [14, 15, 35]. Particularly, in the study by Dalvi and coworkers [35], the changes in the hypothalamus appear to go through a temporary recovery from the effects of the HFD, perhaps due to astrocyte reactivity.

It is well documented that consumption of HFDs can also induce a systemic sub-clinical inflammatory status, which is regarded as an important mediator of whole-body insulin resistance [36]. In this context, it could be argued that the changes in the ME expression of transcripts encoding for inflammatory and BBB proteins could be secondary to diet-induced systemic inflammation. Despite the fact that we did not test this particular question, the timing of events occurring in the ME following the introduction of the HFD precedes by several weeks the emergence of experimental diet-induced systemic inflammation [37], and is in accordance with previous studies evaluating diet-induced hypothalamic inflammation [14, 38].

Next, we evaluated the impact of the consumption of a HFD on the permeability of the BBB in the ME and mediobasal hypothalamus. For that, mice were injected with FITC-dextran, and fluorescence was inspected by confocal microscopy and measured using an ImageJ software. As suspected, there was a rapid increase in the permeability of the ME-BBB, which peaked after 1 week on a HFD, reducing gradually after 2 and 4 weeks. Recent studies have made great advances in the field by identifying ME tanycytes as gatekeepers for nutrients and other peripheral signals that modulate the function of hypothalamic neurons [6, 39]. Tanycytes are specialized glial cells of the hypothalamus that lay in the walls and floor of the third ventricle and act as physical barriers modulating the permeability of fenestrated endothelial cells in the ME [5]. Using transmission electron microscopy, we demonstrated that the cells in the transition between the floor and walls of the third ventricle undergo a major structural disarrangement as early as 1 week after the introduction of the HFD. No previous study has reported such phenomenon; however, it coincides with the very early induction of hypothalamic inflammation by dietary fats [14, 15].

Because the transmission electron microscopy alone is not sufficient to prove that the ME cellular disarrangement promoted by the HFD is in fact due to changes in tanycytes, we performed a series of experiments with confocal microscopy to evaluate the expression and distribution of different proteins involved in the organization of the BBB. We explored the expressions of vimentin, FGF10, and particularly IGFBP2, which is regarded as the most specific marker of β1-tanycytes [4, 39, 40]. We also evaluated the expressions of GLUT1, VEGF and occludin [4, 39, 40], which were not shown in this study because the findings were similar to the results obtained with vimentin, FGF10 and IGFBP2. In general, the immunofluorescence studies revealed that the consumption of a HFD induced a rapid change in the organization and spatial distribution of tanycytes along the floor and wall of the third ventricle and in the transition between the ME and the arcuate nucleus. These changes are particularly evident in the FGF10 labeling in Figs. 4 and 5. The linear distribution was lost, and many cells appeared in a non-organized disposition scattered throughout the mediobasal hypothalamus.

An important finding of this study was that upon persistent consumption of the HFD, the cells expressing IGFBP2 began, after 4 weeks, to express GFAP as well. GFAP is a marker of astrocytes [41]. Studies have shown that, in parallel with microglia [15], astrocytes play an important role in the induction of the hypothalamic inflammatory response in diet-induced obesity [42–44]. Moreover, it has recently been shown that tanycytes can differentiate into astrocytes [45, 46]. Thus, it can be proposed that under prolonged exposure to large portions of dietary fats, tanycytes differentiate into astrocytes, which can mediate at least part of the inflammatory signals that damage the hypothalamic neurons.

In the final part of the study, we explored the hypothesis that BDNF could act as a protective factor against diet-induced ME and hypothalamic damage. Studies have shown that BDNF can attenuate BBB disruption under different conditions [16, 47, 48]. Moreover, acting in the hypothalamus, and particularly through the receptor TrkB, BDNF can attenuate diet-induced obesity through a mechanism related to MC4R signaling [49]. Hypothalamic BDNF can also control other metabolic functions, such as hepatic glucose output and cardiovascular activity [50, 51]. Interestingly, a recent report showed that BDNF is an important determinant of induced pluripotent steam cell (iPSC) differentiation into POMC neurons [52], which suggests that BDNF could play an important role in the putative recovery of the damaged hypothalamus during body mass loss, in the treatment of obesity. Here, we showed that BDNF transcript levels undergo a rapid oscillation following the introduction of a HFD—initially increasing, at 1 week, and then progressively falling to levels that are even lower than those of control after 4 weeks on the HFD. Moreover, the distribution of BDNF and TrkB undergo drastic changes over time during the consumption of a HFD. In lean mice, BDNF is present mostly in the cytoplasm of cells nearby the walls of the third ventricle and shows no co-localization with IGFBP2. Upon consumption of a HFD, BDNF expression occurs predominantly in the perinuclear region of cells scattered throughout the mediobasal hypothalamus; moreover, some cells co-express BDNF and IGFBP2. The immunoneutralization of BDNF in mice fed a HFD resulted in a major phenotypic change, increasing body mass gain, increasing hypothalamic inflammation, and accentuating the already anomalous distribution of IGFBP2-expressing cells. In addition, the immunoneutralization of BDNF was capable of transforming diet-induced obesity-resistant mice in obese-prone mice, which was accompanied by increased expression of inflammatory cytokines in the hypothalamus and increased leakage of the ME-BBB. Taken together the results of our study and previous studies evaluating the effects of BDNF in the hypothalamus, we believe that the immunoneutralization of BDNF reduces whole-body energy expenditure impacting on a number of metabolic functions.

Despite the number of approaches employed to provide a broad view of the main phenomenon described in this study, there are two limitations that should be considered: (i) some of the quantifications were restricted to transcript amount, which may not be always similar to protein amount; (ii) the study did not explore the direct vs. indirect effects of BDNF, since it is known that BDNF has beneficial effects on the metabolic phenotype after HFD feeding and the effects herein described could be a consequence of that and not a direct effect of BDNF on the hypothalamus.

## Conclusions

This is the first report showing that the consumption of large amounts of dietary fats can damage the ME-SFI zone at the functional and structural levels. Moreover, we show that the ME responds to the presence of dietary fats much faster than other CVOs, which may contribute to the anatomical specificity of diet-induced inflammation of the brain. Finally, we identify BDNF as an endogenous ME-BBB protective factor, suggesting that its rapid oscillation (increase followed by an abrupt fall) shortly after the introduction of the dietary fats can play a role in the fragility of the ME.

## Additional files

Additional file 1: Table S1. Quantification of immunofluorescence of Fig. 4. (PDF 67 kb)

Additional file 2: Table S2. Quantification of immunofluorescence of Fig. 5. (PDF 59 kb)

Additional file 3: Table S3. Quantification of immunofluorescence of Fig. 6. (PDF 59 kb)

Additional file 4: Table S4. Quantification of immunofluorescence of Fig. 7d. (PDF 58 kb)

Additional file 5: Table S5. Quantification of immunofluorescence of Fig. 7e. (PDF 59 kb)

Additional file 6: Table S6. Quantification of immunofluorescence of Fig. 8i. (PDF 68 kb)

Additional file 7: Figure S1. Evaluation of the impact of a high-fat diet on the expression of claudin-5 in the median eminence or mice. Six-week-old male Swiss mice were randomly divided to feed on chow or a high-fat diet for 1, 2, or 4 weeks; at the end of the respective experimental periods, the mice were used in experiments. Median eminence was eLaser microdissected for real-time PCR determination of claudin-5. Expression of target transcripts is presented as relative to paired controls fed chow (line in *y* = 1). In all conditions, *n* = 4; *p < 0.05 vs. respective control. W, week. (PDF 140 kb)

Additional file 8: Figure S2. Evaluation of the blood-brain barrier integrity. The protocol employed for evaluation of BBB integrity is shown in Fig. 2a. Confocal microscopy analysis was employed for determining FITC-dextran endogenous fluorescence in the regions of the vascular organ of lamina terminalis (OVLT), subfornical organ (SFO), and subcomissural organ (SCO); in all acquisitions, the same settings of the microscope were employed (laser 488, wavelength = 405, %laser = 20%, gain = 1015, offset = − 0.3799). The fluorescence in the distinct regions was determined using an ImageJ software and presented as relative to control. *N* = 4. (PDF 141 kb)

Additional file 9: Figure S3. Immunofluorescence staining of markers of the median eminence blood-brain barrier and markers of glial cells. Immunofluorescence staining using primary antibodies against IGFBP2 (green), GFAP (red) (A), and IBA1 (red) (B). Specimens were obtained from bregma-anteroposterior − 2.06 to − 2.18. The images are representative of three independent experiments. 3v, third ventricle; IGFBP2, insulin-like growth factor-binding protein 2; GFAP, glial fibrillary acidic protein; IBA1, ionized calcium-binding adapter molecule-1. (PDF 17432 kb)

Additional file 10: Figure S4. Evaluation of median eminence blood-brain barrier integrity in mice treated with an anti-BDNF immunoneutralizing antibody. The protocol employed for evaluation of BBB integrity is shown in Fig. 2a. Confocal microscopy analysis was employed for determining FITC-dextran endogenous fluorescence in the region of the median eminence in all acquisitions; the same settings of the microscope were employed (laser 488, wavelength = 405, %laser = 20%, gain = 1015, offset = − 0.3799). The fluorescence intensity was determined using an ImageJ software and presented as relative to control. *N* = 6. ABDNF, antibody-anti-BDNF; BDNF, brain-derived neurotrophic factor; CTR, control; HFD, high-fat diet; IGG, non-immune antiserum. (PDF 2780 kb)
