# Supplementary material for: Dietary fats promote functional and structural changes in the median eminence blood/spinal fluid interface—the protective role for BDNF
Source: J Neuroinflammation. 2018 Jan 9;15:10. doi: 10.1186/s12974-017-1046-8 (PMC5761204; doi:10.1186/s12974-017-1046-8)
Supplement: Supplementary file 1 — Quantification of immunofluorescence of Fig. 4. (PDF 67 kb) [file 12974_2017_1046_MOESM1_ESM.pdf]

JNEU-D-17-00210 R1

Research Article

# Dietary fats promote functional and structural changes in the median eminence blood/spinal fluid interface - The protective role for BDNF

Albina F. Ramalho[1], Bruna Bombassaro[1], Nathalia R. Dragano[1], Carina Solon[1], Joseane Morari[1], Milena Fioravante[1], Roberta Barbizan[1], Licio A. Velloso[1*], Eliana P. Araujo[2]

## Supplementary Data

**Supplementary Table 1. Quantification of immunofluorescence of Figure 4.**

| | | | CTR_VIM | HFD_1S | HFD_2S | HFD_4S |
|---|---|---|---|---|---|---|
| **VIM+FGF** | **VIM** | **Mean** | 9,80 | 8,00 | 8,21 | 9,08 |
| | | **SD** | 2,28 | 0,96 | 1,05 | 2,40 |
| | **FGF** | **Mean** | 6,04 | 4,48 | 4,63 | 5,74 |
| | | **SD** | 5,32 | 4,98 | 5,06 | 4,72 |
