# Supplementary material for: Dietary fats promote functional and structural changes in the median eminence blood/spinal fluid interface—the protective role for BDNF
Source: J Neuroinflammation. 2018 Jan 9;15:10. doi: 10.1186/s12974-017-1046-8 (PMC5761204; doi:10.1186/s12974-017-1046-8)
Supplement: Supplementary file 3 — Quantification of immunofluorescence of Fig. 6. (PDF 59 kb) [file 12974_2017_1046_MOESM3_ESM.pdf]

JNEU-D-17-00210 R1

Research Article

Dietary fats promote functional and structural changes in the median eminence blood/spinal fluid interface - The protective role for BDNF

Albina F. Ramalho[1], Bruna Bombassaro[1], Nathalia R. Dragano[1], Carina Solon[1], Joseane Morari[1], Milena Fioravante[1], Roberta Barbizan[1], Licio A. Velloso[1*], Eliana P. Araujo[2]

Supplementary Data

Supplementary Table 3. Quantification of immunofluorescence of Figure 6.

**IGFBP2+GFAP**	**IGFBP2**	**Mean**	5,68	4,73	4,84	5,23
		**SD**	0,51	0,35	0,31	0,72
	**GFAP**	**Mean**	3,09	2,54	2,58	2,98
		**SD**	3,65	3,09	3,21	3,19
