# Supplementary material for: Dietary fats promote functional and structural changes in the median eminence blood/spinal fluid interface—the protective role for BDNF
Source: J Neuroinflammation. 2018 Jan 9;15:10. doi: 10.1186/s12974-017-1046-8 (PMC5761204; doi:10.1186/s12974-017-1046-8)
Supplement: Supplementary file 4 — Quantification of immunofluorescence of Fig. 7d. (PDF 58 kb) [file 12974_2017_1046_MOESM4_ESM.pdf]

JNEU-D-17-00210 R1

Research Article

Dietary fats promote functional and structural changes in the median eminence blood/spinal fluid interface - The protective role for BDNF

Albina F. Ramalho[1], Bruna Bombassaro[1], Nathalia R. Dragano[1], Carina Solon[1], Joseane Morari[1], Milena Fioravante[1], Roberta Barbizan[1], Licio A. Velloso[1*], Eliana P. Araujo[2]

Supplementary Data

Supplementary Table 4. Quantification of immunofluorescence of Figure 7D.

**IGFBP2+BDNF**	**IGFBP2**	**Mean**	1,99	1,66
		**SD**	0,16	0,08
	**BDNF**	**Mean**	1,08	0,87
		**SD**	1,30	1,12
