# Supplementary material for: Dietary fats promote functional and structural changes in the median eminence blood/spinal fluid interface—the protective role for BDNF
Source: J Neuroinflammation. 2018 Jan 9;15:10. doi: 10.1186/s12974-017-1046-8 (PMC5761204; doi:10.1186/s12974-017-1046-8)
Supplement: Supplementary file 5 — Quantification of immunofluorescence of Fig. 7e. (PDF 59 kb) [file 12974_2017_1046_MOESM5_ESM.pdf]

JNEU-D-17-00210 R1

Research Article

# Dietary fats promote functional and structural changes in the median eminence blood/spinal fluid interface - The protective role for BDNF

Albina F. Ramalho[1], Bruna Bombassaro[1], Nathalia R. Dragano[1], Carina Solon[1], Joseane Morari[1], Milena Fioravante[1], Roberta Barbizan[1], Licio A. Velloso[1*], Eliana P. Araujo[2]

## Supplementary Data

**Supplementary Table 5. Quantification of immunofluorescence of Figure 7E.**

| | | | | |
|---|---|---|---|---|
| **IGFBP2+TRKb** | **TrkB** | **Mean** | 1,19 | 0,99 |
| | | **SD** | 0,16 | 0,18 |
| | **IGFBP2** | **Mean** | 0,67 | 0,58 |
| | | **SD** | 0,73 | 0,58 |
