# Supplementary material for: Dietary fats promote functional and structural changes in the median eminence blood/spinal fluid interface—the protective role for BDNF
Source: J Neuroinflammation. 2018 Jan 9;15:10. doi: 10.1186/s12974-017-1046-8 (PMC5761204; doi:10.1186/s12974-017-1046-8)
Supplement: Supplementary file 6 — Quantification of immunofluorescence of Fig. 8i. (PDF 68 kb) [file 12974_2017_1046_MOESM6_ESM.pdf]

JNEU-D-17-00210 R1

Research Article

Dietary fats promote functional and structural changes in the median eminence blood/spinal fluid interface - The protective role for BDNF

Albina F. Ramalho[1], Bruna Bombassaro[1], Nathalia R. Dragano[1], Carina Solon[1], Joseane Morari[1], Milena Fioravante[1], Roberta Barbizan[1], Licio A. Velloso[1*], Eliana P. Araujo[2]

Supplementary Data

Supplementary Table 6. Quantification of immunofluorescence of Figure 8I.

			CTR_IgG	CTR_ABDNF	HFD_IgG	HFD_ABDNF
**TRAT_ABDNF**	**IGFBP2**	**Mean**	14,81	18,38	10,75	7,74
		**SD**	7,57	10,29	1,21	2,87
	**BDNF**	**Mean**	19,06	14,02	13,78	8,89
		**SD**	10,45	7,45	9,30	4,03
