# Supplementary material for: Dietary fats promote functional and structural changes in the median eminence blood/spinal fluid interface—the protective role for BDNF
Source: J Neuroinflammation. 2018 Jan 9;15:10. doi: 10.1186/s12974-017-1046-8 (PMC5761204; doi:10.1186/s12974-017-1046-8)

JNEU-D-17-00210 R1

Research Article

Dietary fats promote functional and structural changes in the median eminence blood/spinal fluid interface - The protective role for BDNF

Albina F. Ramalho[1], Bruna Bombassaro[1], Nathalia R. Dragano[1], Carina Solon[1], Joseane Morari[1], Milena Fioravante[1], Roberta Barbizan[1], Licio A. Velloso[1*], Eliana P. Araujo[2]

Supplementary Data

Supplementary Figure 1. Evaluation of the impact of a high-fat diet on the expression of Claudin-5 in the median eminence or mice. Six-week old, male, Swiss mice were randomly divided to feed on chow or a high-fat diet for one, two or four weeks; at the end of the respective experimental periods, the mice were used in experiments. Median eminence was e laser microdissected for real-time PCR determination of claudin-5. Expression of target transcripts is presented as relative to paired controls fed chow (line in y=1). In all conditions n=4; *p<0.05 *vs.* respective control. W, week.

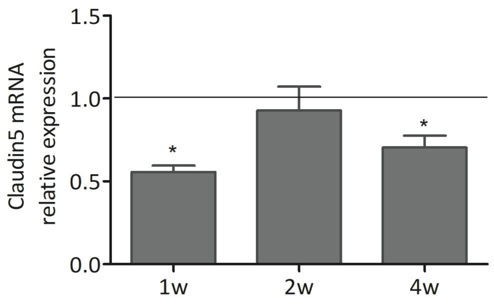

Supplement: Supplementary file 7 — Evaluation of the impact of a high-fat diet on the expression of claudin-5 in the median eminence or mice. Six-week-old male Swiss mice were randomly divided to feed on chow or a high-fat diet for 1, 2, or 4 weeks; at the end of the respective experimental periods, the mice were used in experiments. Median eminence was eLaser microdissected for real-time PCR determination of claudin-5. Expression of target transcripts is presented as relative to paired controls fed chow (line in y = 1). In all conditions, n = 4; *p < 0.05 vs. respective control. W, week. (PDF 140 kb) [file 12974_2017_1046_MOESM7_ESM.pdf]
