# Supplementary material for: Dietary fats promote functional and structural changes in the median eminence blood/spinal fluid interface—the protective role for BDNF
Source: J Neuroinflammation. 2018 Jan 9;15:10. doi: 10.1186/s12974-017-1046-8 (PMC5761204; doi:10.1186/s12974-017-1046-8)

JNEU-D-17-00210 R1

Research Article

Dietary fats promote functional and structural changes in the median eminence blood/spinal fluid interface - The protective role for BDNF

Albina F. Ramalho[1], Bruna Bombassaro[1], Nathalia R. Dragano[1], Carina Solon[1], Joseane Morari[1], Milena Fioravante[1], Roberta Barbizan[1], Licio A. Velloso[1*], Eliana P. Araujo[2]

Supplementary Data

Supplementary Figure 2. Evaluation of the blood-brain barrier integrity. The protocol employed for evaluation of BBB integrity is shown in Figure 2A. Confocal microscopy analysis was employed for determining FITC-dextran endogenous fluorescence in the regions of the vascular organ of lamina terminalis (OVLT), subfornical organ (SFO) and subcomissural organ (SCO); in all acquisitions, the same settings of the microscope were employed (laser 488, wave-length=405, %laser=20%, gain=1015, offset=-0.3799). The fluorescence in the distinct regions was determined using Image-J software and presented as relative to control. N=4.

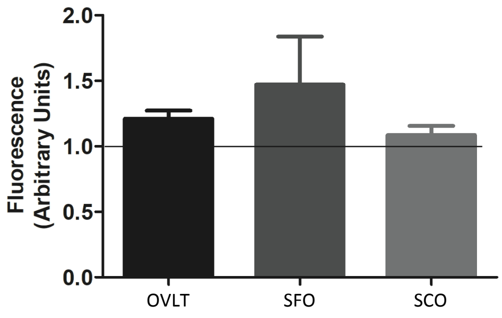

Supplement: Supplementary file 8 — Evaluation of the blood-brain barrier integrity. The protocol employed for evaluation of BBB integrity is shown in Fig. 2a. Confocal microscopy analysis was employed for determining FITC-dextran endogenous fluorescence in the regions of the vascular organ of lamina terminalis (OVLT), subfornical organ (SFO), and subcomissural organ (SCO); in all acquisitions, the same settings of the microscope were employed (laser 488, wavelength = 405, %laser = 20%, gain = 1015, offset = − 0.3799). The fluorescence in the distinct regions was determined using an ImageJ software and presented as relative to control. N = 4. (PDF 141 kb) [file 12974_2017_1046_MOESM8_ESM.pdf]
