# Supplementary material for: Dietary fats promote functional and structural changes in the median eminence blood/spinal fluid interface—the protective role for BDNF
Source: J Neuroinflammation. 2018 Jan 9;15:10. doi: 10.1186/s12974-017-1046-8 (PMC5761204; doi:10.1186/s12974-017-1046-8)

JNEU-D-17-00210 R1

Research Article

Dietary fats promote functional and structural changes in the median eminence blood/spinal fluid interface - The protective role for BDNF

Albina F. Ramalho[1], Bruna Bombassaro[1], Nathalia R. Dragano[1], Carina Solon[1], Joseane Morari[1], Milena Fioravante[1], Roberta Barbizan[1], Licio A. Velloso[1*], Eliana P. Araujo[2]

Supplementary Data

Supplementary Figure 3. Immunofluorescence staining of markers of the median eminence blood-brain barrier and markers of glial cells. Immunofluorescence staining using primary antibodies against IGFBP2 (green), GFAP (red) (A) and IBA1 (red) (B). Specimens were obtained from bregma-anteroposterior -2,06 to -2,18 from mice fed on a high-fat diet (HFD) and treated with either a pre-immune antiserum (IgG) or with an anti-BDNF antibody (BDNF). The images are representative of four independent experiments. 3v, third ventricle; BDNF, brain-derived neurotrophic factor IGFBP2, insulin-like growth-factor binding protein 2; GFAP, glial fibrillary acidic protein. IBA1, ionized calcium-binding adapter molecule-1.

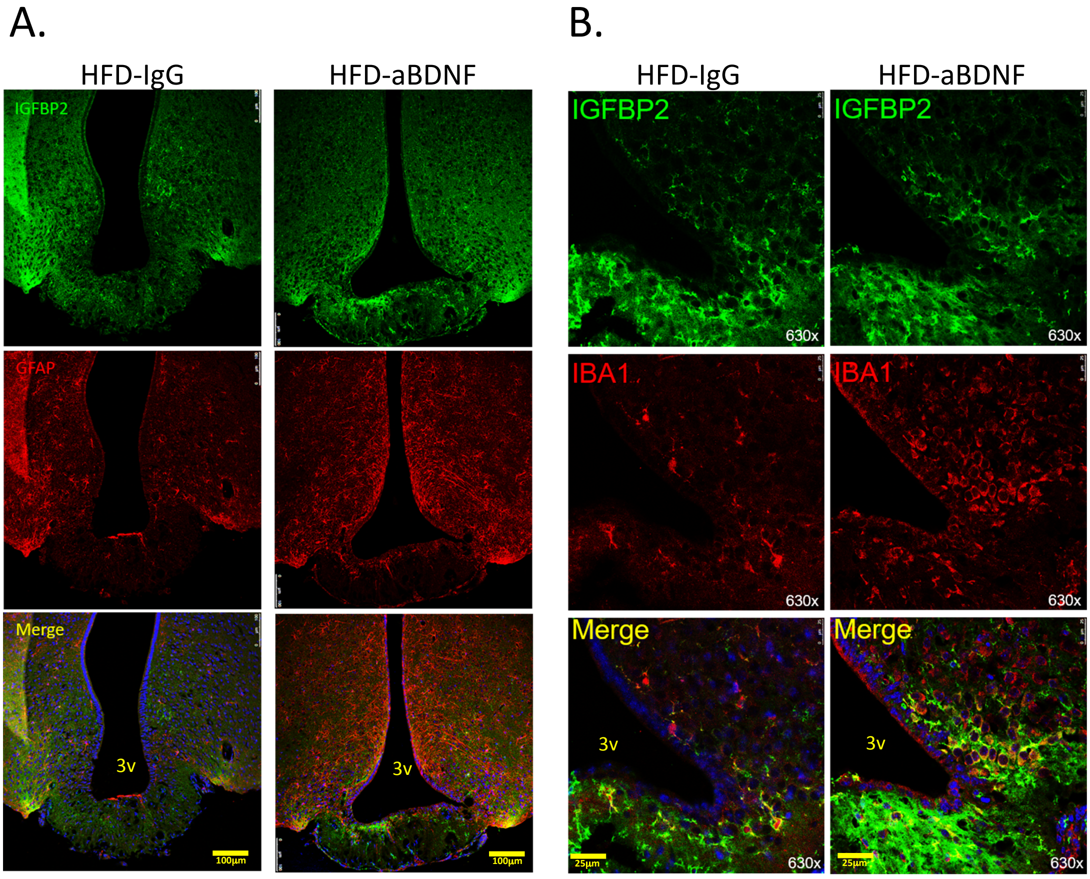

Supplement: Supplementary file 9 — Immunofluorescence staining of markers of the median eminence blood-brain barrier and markers of glial cells. Immunofluorescence staining using primary antibodies against IGFBP2 (green), GFAP (red) (A), and IBA1 (red) (B). Specimens were obtained from bregma-anteroposterior − 2.06 to − 2.18. The images are representative of three independent experiments. 3v, third ventricle; IGFBP2, insulin-like growth factor-binding protein 2; GFAP, glial fibrillary acidic protein; IBA1, ionized calcium-binding adapter molecule-1. (PDF 17432 kb) [file 12974_2017_1046_MOESM9_ESM.pdf]
