# Supplementary material for: Dietary fats promote functional and structural changes in the median eminence blood/spinal fluid interface—the protective role for BDNF
Source: J Neuroinflammation. 2018 Jan 9;15:10. doi: 10.1186/s12974-017-1046-8 (PMC5761204; doi:10.1186/s12974-017-1046-8)

JNEU-D-17-00210 R1

Research Article

Dietary fats promote functional and structural changes in the median eminence blood/spinal fluid interface - The protective role for BDNF

Albina F. Ramalho[1], Bruna Bombassaro[1], Nathalia R. Dragano[1], Carina Solon[1], Joseane Morari[1], Milena Fioravante[1], Roberta Barbizan[1], Licio A. Velloso[1*], Eliana P. Araujo[2]

Supplementary Data

Supplementary Figure 4. Evaluation of median eminence blood-brain barrier integrity in mice treated with an anti-BDNF immunoneutralizing antibody. The protocol employed for evaluation of BBB integrity is shown in Figure 2A. Confocal microscopy analysis was employed for determining FITC-dextran endogenous fluorescence in the region of the median eminence in all acquisitions, the same settings of the microscope were employed (laser 488, wave-length=405, %laser=20%, gain=1015, offset=-0.3799). The fluorescence intensity was determined using Image-J software and presented as relative to control. N=6. ABDNF, antibody-anti-BDNF; BDNF, brain-derived neurotrophic factor; CTR, control; HFD, high-fat diet; IGG, non-immune antiserum.

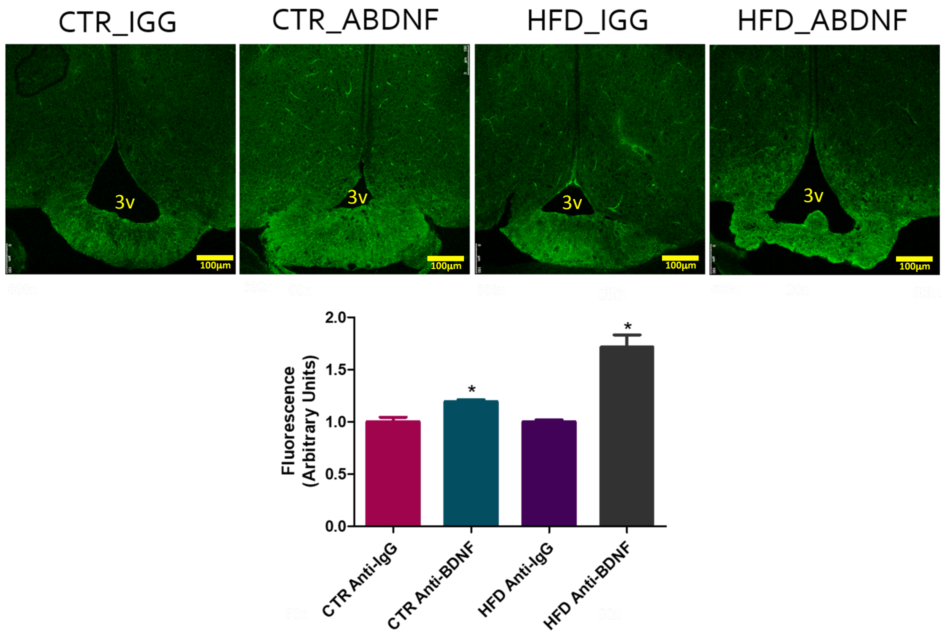

Supplement: Supplementary file 10 — Evaluation of median eminence blood-brain barrier integrity in mice treated with an anti-BDNF immunoneutralizing antibody. The protocol employed for evaluation of BBB integrity is shown in Fig. 2a. Confocal microscopy analysis was employed for determining FITC-dextran endogenous fluorescence in the region of the median eminence in all acquisitions; the same settings of the microscope were employed (laser 488, wavelength = 405, %laser = 20%, gain = 1015, offset = − 0.3799). The fluorescence intensity was determined using an ImageJ software and presented as relative to control. N = 6. ABDNF, antibody-anti-BDNF; BDNF, brain-derived neurotrophic factor; CTR, control; HFD, high-fat diet; IGG, non-immune antiserum. (PDF 2780 kb) [file 12974_2017_1046_MOESM10_ESM.pdf]
